# Relating structure and composition with accessibility of a single catalyst particle using correlative 3-dimensional micro-spectroscopy

**DOI:** 10.1038/ncomms12634

**Published:** 2016-08-30

**Authors:** Yijin Liu, Florian Meirer, Courtney M. Krest, Samuel Webb, Bert M. Weckhuysen

**Affiliations:** 1 Stanford Synchrotron Radiation Lightsource, SLAC National Accelerator Laboratory, Menlo Park, California, 2575 Sand Hill Road, Menlo Park, California 94025, USA; 2 Inorganic Chemistry and Catalysis group, Debye Institute for Nanomaterials Science, Utrecht University, Utrecht, Universiteitsweg 99, 3584 CG Utrecht, The Netherlands

## Abstract

To understand how hierarchically structured functional materials operate, analytical tools are needed that can reveal small structural and chemical details in large sample volumes. Often, a single method alone is not sufficient to get a complete picture of processes happening at multiple length scales. Here we present a correlative approach combining three-dimensional X-ray imaging techniques at different length scales for the analysis of metal poisoning of an individual catalyst particle. The correlative nature of the data allowed establishing a macro-pore network model that interprets metal accumulations as a resistance to mass transport and can, by tuning the effect of metal deposition, simulate the response of the network to a virtual ageing of the catalyst particle. The developed approach is generally applicable and provides an unprecedented view on dynamic changes in a material's pore space, which is an essential factor in the rational design of functional porous materials.

## Abstract (web summary)

The performance of catalytic materials is determined by small-scale chemical and structural variations over large volumes. Here the authors report a correlative spectroscopy approach capable of visualizing processes over multiple length scales, and model the effects of poisoning on mass transport.

## Graphical abstract

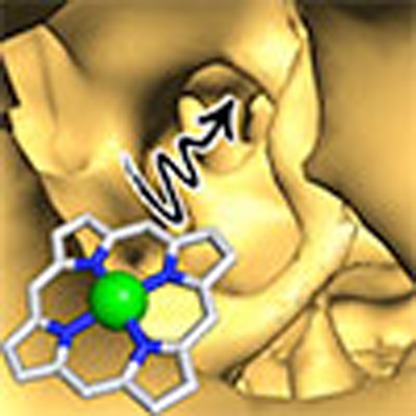

A key aspect in the rational design of functional materials, such as catalysts, batteries, fuels cells and optical devices, is the interplay of processes that happen at different length scales. This is because the macroscopic behaviour of materials is largely determined by the arrangement of its nano- and micro-scale building blocks. Although modern advanced imaging tools have sufficient resolving power to investigate very fine structures in a small sub-volume of a material, it remains challenging to probe processes that happen at the nanometre scale across micrometre-length scales. Furthermore, because chemical processes are studied, tools are needed that provide sufficient contrast for both sample morphology and local chemistry. This is particularly and exemplarily the case when studying the complex processes that take place in materials used in heterogeneous catalysis. A typical example for such a mesoscale process is the metal poisoning of fluid catalytic cracking (FCC) catalysts.

In the FCC process, the residue that remains after distilling crude oil and that contains large and heavy hydrocarbon molecules is converted (‘cracked') into lighter, more valuable fractions like gasoline and propylene^1^,^2^,^3^. The FCC catalyst is designed as a multi-component, hierarchically porous particle of 50–100 μm diameters and consists of catalytically highly active phases (zeolites) that are embedded in a matrix consisting of an active component (alumina) and a non-active part made from silica and clay^2^,^3^,^4^. During FCC operation, the particles are exposed to harsh conditions (high temperature) and the non-active components of the catalyst particle are added to increase the attrition resistance of the catalyst as well as the hydrothermal stability of the zeolite material^5^,^6^,^7^. The latter is further enhanced by incorporating rare-earth metals, such as La, Ce and Pr, into the zeolite^8^,^9^,^10^,^11^. The actual cracking of the feedstock molecules happens through pre-cracking and cracking in the particles' active alumina and zeolite phases, respectively^3^,^12^,^13^. The active phases, therefore, need to be accessible for feedstock molecules and the cracking products need to be able to leave the catalyst. This is guaranteed by a highly interconnected hierarchical pore-network in the catalyst with pore sizes ranging from micro-pores^14^ (< 2 nm pore diameter) in the zeolite phase, to meso-14 (<50 nm) and macro-pores^14^ (>50 nm) in the active alumina phase and matrix^7^,^15^,^16^,^17^. The pore space and its interconnectivity is thus of key importance to the efficiency of the conversion process, and recently we have shown that deposition of Fe and Ni can cause an irreversible blockage and restriction of the available pore space that is directly related to a decrease in catalyst cracking efficiency^18^,^19^.

The term metal poisoning summarizes a range of physicochemical effects that become a serious issue for cracking efficiency when the processed feedstock contains elevated levels of any of the known poisonous metals Fe, Ni, V, Ca, Cu and Na^8^,^20^,^21^,^22^,^23^,^24^,^25^,^26^,^27^. Known sources of these metals in the feedstock are metal contaminations already present in the crude oil and/or originating from upstream processes and equipment. These effects can be divided into two groups: damage (direct or indirect) to the catalytically most active zeolite domains in the FCC particle, and a reduction of particle accessibility. When studying these effects, it is important to investigate real-life catalysts from an industrial FCC unit because it has been proven difficult (if not simply impossible) for lab-based deactivation methods to accurately mimic the effects that occur during the industrial FCC process^9^,^28^. To obtain a complete picture of an individual catalyst particle's pore-network and related catalyst accessibility, it is essential to study the distribution of the accumulated metals within the entire catalyst particle in three dimensions.

Recent studies of FCC catalyst particles using variants of hard X-ray nano-tomography showed that the spatial resolution of synchrotron-based X-ray imaging has approached the interface between the macro- and meso-pore regimes^18^,^19^,^20^,^29^,^30^,^31^,^32^,^33^, which means that pore sizes around 50 nm start to become visible. In particular, hard X-ray full-field transmission X-ray microscopy (TXM) has emerged as a suitable method as it allows investigating whole particles and can combine information about particle morphology with three-dimensional (3D) elemental concentration distributions. However, TXM measurements of multiple elemental distributions are limited in terms of sensitivity and flexibility, mainly due to restrictions by X-ray optics, which limited previous studies to the investigation of Fe and Ni.

One possibility to improve sensitivity for elemental detection is to use hard X-ray micro/nano focusing techniques in XRF detection mode, which provides the information of multiple elements of interest simultaneously. In this mode, the spatial resolution is limited by the beam spot size used to raster scan the sample, that is, the ability to focus X-rays. Advanced X-ray focusing techniques^34^,^35^ have made it possible to perform this type of XRF mapping at different length scales ranging from the micron^36^,^37^ down to the nanometre level^29^,^38^,^39^,^40^,^41^. However, the mechanical raster scan becomes very time consuming for covering a large field of view with a small beam spot, especially when tomography is desired. As a result, XRF tomography of whole FCC catalyst particles is usually limited by the measuring time and does not provide high-resolution morphological information about the material's pore space.

In this work, a correlative 3D micro-spectroscopic approach combining the methods of nano-TXM and multiple-element micro X-ray fluorescence (μ-XRF) tomography has been developed. The correlative measurement provided both the 3D morphology at sub-100 nm 3D resolution and sufficient chemical sensitivity to map the 3D distribution of multiple elements with micrometre resolution. On the basis of this correlative data set, we developed a detailed analysis approach studying the effect of metal deposition on the macro-pore network stability of a whole, single FCC particle obtained from an industrial plant. Our approach allowed testing the response of the particle's pore network to a gradually increasing blockage of the pore channels by the poisoning metals. This effectively simulates catalytic ageing of the pore network that goes along with a decreasing cracking efficiency of the catalyst.

## Results

### Correlative imaging of an individual FCC particle

**Figure 1:**
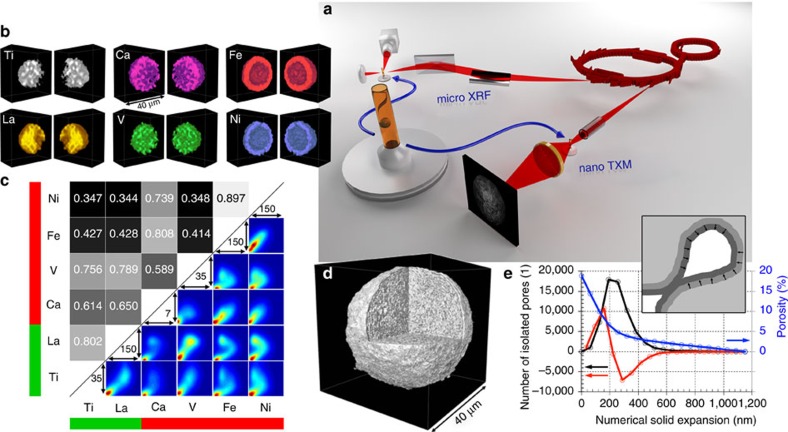
Correlative three-dimensional X-ray micro-spectroscopy. (**a**) Hard X-rays emitted by two different insertion devices at the storage ring of the Stanford Synchrotron Radiation Lightsource (SSRL) were used for μ-XRF and nano-TXM tomography of an individual FCC catalyst particle that was loaded in a Kapton capillary with 200 μm inner diameter and 20 μm wall thickness. (**b**,**c**) In **b**, the 3D distributions of all elements in the FCC particle are presented that showed relevant count rates (intensities were corrected for self-absorption effects, see Supplementary Methods: correction of self-absorption effects in micro XRF tomography and Supplementary Fig. 4). These data were then used in the analysis of the spatial correlation of all elements of interest using Pearson's correlation coefficient (PCC) and the correlation plots reported in **c**. (**d**) High resolution TXM tomography data revealing the internal and external structure of the particle that is virtually cut open (see also Supplementary Movie 1). These data were then used in a pore throat analysis (**e**) applying a numerical solid expansion approach^45^.

Figure 1 shows a schematic of the experimental setup (a) and the data obtained by the correlated approach (b–e). Although μ-XRF tomography measured 3D distributions of multiple elements at lower resolution (determined by the size of the X-ray focal spot of 2 μm), nano-TXM tomography provided morphological information of the same catalyst particle at a 3D resolution of 98.2 nm (see the ‘Methods' section: X-ray nano tomography and Supplementary Fig. 1). This allowed correlating pore space morphology with distribution and relative concentration of the elements (Supplementary Figs 2 and 3), which are grouped into metal poisons (Fe, Ni, V and Ca) and markers for structural components (Ti in the clay and La in the rare-earth exchanged ultra-stable Y (USY) zeolites). Fe is present as both, poison and part of the clay component, however, several studies have shown that the high Fe concentration found at the surface of FCC particles is not present in a fresh catalyst and can unambiguously be related to Fe poisoning^18^,^19^,^20^,^21^,^22^,^41^. The results clearly show that Fe, Ni and Ca are preferentially accumulated in/on the catalyst particle surface, while V was found heterogeneously distributed throughout the FCC particle (Fig. 1b).

Similarities in the spatial distributions were quantified using bimodal spatial correlation analyses for all element pairs (Fig. 1c). The correlation plot for each pair is displayed as a heat map visualizing the ratios of the respective elemental concentrations of all particle voxels. The intensity ranges (in counts) used for the *x* and *y* axes of each correlation plot are the same as in Supplementary Fig. 4 and the colour ranges indicate the number of voxels with same elemental ratio from dark blue (a single voxel) to dark red (many voxels). The upper left part of Fig. 1c shows the correlation expressed by Pearson's linear correlation coefficient (PCC), which can have values between –1 and 1. The closer the value is to plus or minus one the higher is the degree of positive or negative correlation, while a value of zero indicates no correlation. In the plot, the value of the PCC is reflected as a grey scale level, ranging from the lowest (black) to the highest correlation (white). The green and red bars indicate structural markers (Ti and La) and metal poisons (Ni, Fe, V, Ca), respectively.

These data suggest a similar accumulation mechanism for Fe, Ni and Ca, while V is either deposited via a different mechanism or shows a higher mobility after deposition as previously suggested^9^,^27^,^42^. The correlation between V, La and Ti can be attributed to the fact that Ti can act as a V trap^43^,^44^ and/or the limited spatial resolution of the μ-XRF data, which prevents a clear separation of the La-containing zeolite domains (<2 μm in size) from the clay component (kaolin) that contains Ti mainly in the form of rutile^25^.

In panels d and e of Fig. 1, the morphological information obtained by nano-TXM is displayed. The particle appears to be a typical FCC catalyst particle when compared with previous studies^18^,^19^,^20^,^21^,^29^,^41^: it has a diameter of about 40 μm, shows a denser surface layer of 1–2 μm thickness and a complex macro-pore structure throughout the rest of the particle; the total porosity was determined to 18.8%. In this study, we additionally performed a pore throat analysis^45^, which revealed a pore throat size distribution peak at about 320 nm. The plot in panel e reports how porosity (blue line) and the number of isolated pores (black line) change as the solid phase (light grey area in the inset) is numerically grown layer by layer as schematically depicted in the inset. Layers of single voxel (64 nm) thickness are represented in dark grey. The peak in the derivative (red line) of the number of isolated pores indicates that most pore throats get closed at an expansion layer thickness of ∼160 nm. This dominance of macro-pore channels with diameters above 100 nm underlines their important function as ‘molecular highways' of the catalyst particle's pore network, which connects the interior with the exterior of the particle for feedstock molecules to enter and product molecules to exit after cracking.

### Modelling of the pore network's response to metal poisoning

**Figure 2:**
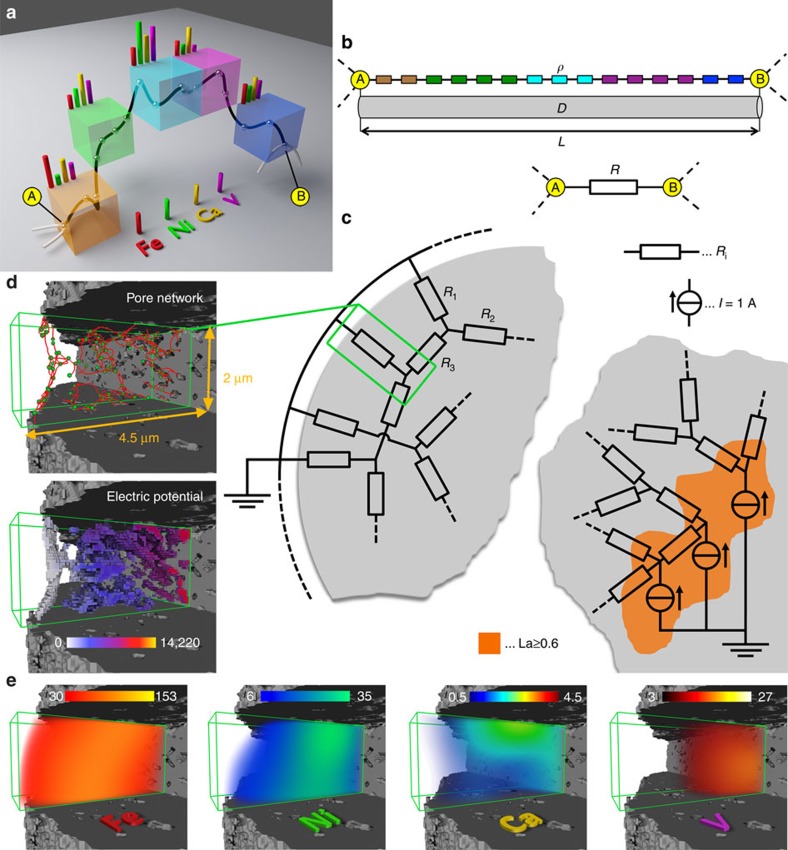
Establishing the resistor network from 3D μ-XRF and nano-TXM tomography data. (**a**) Schematic of a pore channel (black) connecting nodes A and B that represents the pore space passing through 5 volume units with distinct elemental concentrations. (**b**) In analogy to a conducting wire, this connection can be represented by its length *L*, the average pore channel cross section *D* and a specific resistivity *ρ*, defined as one plus the summed average concentrations of poisoning metals along the pore channel. (**c**) Every pore channel of the studied FCC particle can be expressed by a resistor with a resistivity defined as *R*=*ρ* × *L*/*D*. Then nodes of the pore network located in regions of elevated La concentrations were defined as current sources of 1 A, while entry nodes at the particle surface were set to ground potential (0 V). (**d**) An arbitrarily selected sub-volume located near the surface of the particle and the corresponding pseudo-electric potential (in volts) as calculated using nodal analysis of the established virtual resistor network. (**e**) The poisoning metals' μ-XRF signal over the same sub-volume, which were used to determine the specific resistance as schematically depicted in **a** and **b** (colour scales report μ-XRF counts).

In the next step, this pore network was analysed in detail considering both pore connectivity and the correlated 3D elemental distributions. The combination of data from nano-TXM and μ-XRF tomography allowed an assessment of the degree of pore blockage or ‘resistance' that the metal poisons Fe, Ni, Ca and V pose to mass transport through the pore network. First the pore space reconstructed from nano-TXM was skeletonized, resulting in a set of points and connecting cylinders with diameters that correspond to the determined pore diameters^19^. Then, using La as a structural marker for zeolites, regions of elevated La XRF intensities (≥60% of the maximum La intensity) were defined as the active regions that should be connected to the exterior of the particle (see Supplementary Methods: Resistor network model). Therefore, all nodes of the pore network located within these zeolite domains were labelled as ‘source nodes', while all nodes at the surface of the FCC particle were labelled ‘sink nodes'. To assess how easily molecules can travel between source and sink nodes, we used the analogy of an electrical resistor network^46^,^47^,^48^. Figure 2 shows schematically how this pseudo-resistor network was established on the basis of the measured macro-pore network and the elemental concentration distributions of La, Fe, Ni, Ca and V.

**Figure 3:**
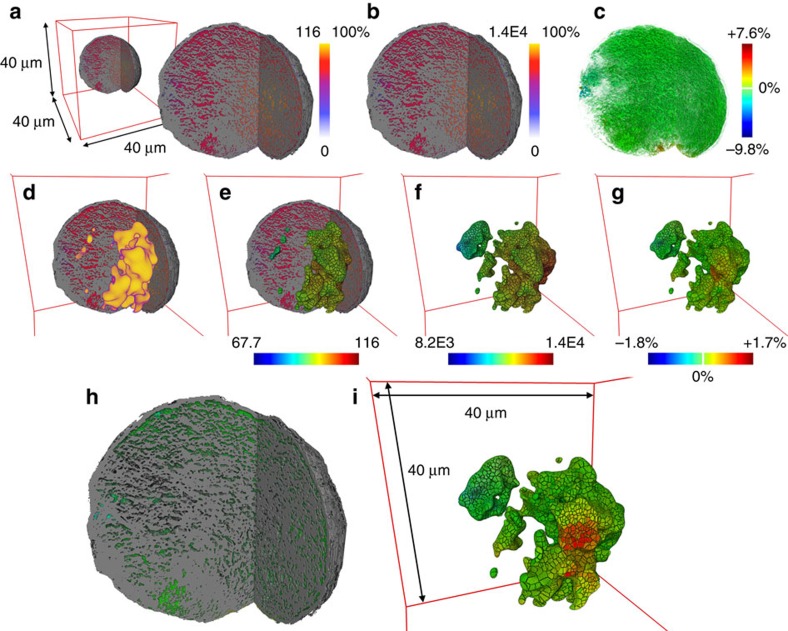
Simulated 3D potential and equivalent resistance distribution in the catalyst particle. Potential and equivalent resistance values were determined by nodal analysis of the resistor network that was established from the measured macro-pore network and the metal distribution in the FCC particle under study. (**a**,**b**) Show a virtual cut through the X-ray nano-TXM data and the pseudo-electric potential in the macro-pores determined (**a**) without considering and (**b**) considering an increased specific resistance by the metals Fe, Ni, Ca and V. (**c**–**i**)To exclusively inspect changes in the pseudo-voltage distribution, **c** and **h** report the difference between the normalized (0–100%) distributions shown in **a** and **b**. The equivalent resistance determined for each of the source nodes that were identified via the La concentration distribution in the FCC particle (**d**) and using the potentials shown in **a** and **b** is visualized in **e** and **f**, respectively. The difference of normalized equivalent resistance distributions is reported in **g** and **i** (same colour bar). In the latter, a virtual cut through the data reveals some of the domains that show the strongest changes in equivalent resistance.

The established resistor network was used to calculate an electric potential in every node using nodal analysis and an equivalent resistance for each source node applying Ohm's law. Following the analogy between an electrical resistor network and the flow in the network model of a porous medium, the calculated potential can be interpreted as the pressure of the flowing medium. The equivalent resistor value obtained for each source node indicates how difficult it is for feedstock molecules to reach this node (that is, zeolite domain) from the surface. Here it is important to note that we consider the whole complexity of the macro-pore network, namely pore geometries and interconnectivity. The equivalent resistance distribution shows that although the macro pore network is highly interconnected, not all regions of elevated La concentrations (that is, zeolite domains) are equally accessible (Fig. 3).

The 3D distribution of the potential over the resistor network is displayed in Fig. 3 highlighting the heterogeneity of the particle's accessibility. To assess the effect of the metals and their distribution, the calculation was performed twice, first using a value of one as a specific resistance for all resistors and then using an individually determined value for each resistor, namely one plus the sum of the XRF intensities of Fe, Ni, Ca and V along the respective pore channel (see Supplementary Methods: Resistor network model). Here the model assumes that each metal increases resistance to the same extent, because the actual degree of pore blocking and its relation to the measured XRF intensities of the elements is unknown. In this study, we focused on the effect of the total metal loading, however, a weighting based on a possible element specific degree of resistance can be easily implemented once it is determined. The results showed that the local changes in specific resistance caused by the poisoning metals increase the absolute pseudo-voltage and equivalent resistance, but do not significantly change the distributions of simulated potential (<10%) and equivalent resistance (<2%). The reason is the high interconnectivity of the network that largely compensates any influence of an increased specific resistance in spatially confined parts of the network.

In the previous step, a complete pore blockage (that is, pore channels with infinite resistance) was not considered, because it is unknown at which threshold of metal concentrations a pore channel gets completely blocked. Therefore, to test the stability of the network against pore blockage by the poisoning metals, we used not one but a series of 73 resistance thresholds ranging from 8 to 80 Ohm to remove resistors with a resistance larger than the selected threshold, simulating complete pore blockage of these specific pore channels (see sections ‘Resistor network model' and ‘Re-evaluating the interconnectivity of the pore network' in the Supplementary Methods). Varying this threshold, therefore, acts like a slider for time, simulating a gradual increase in the effect of the deposited metal.

**Figure 4:**
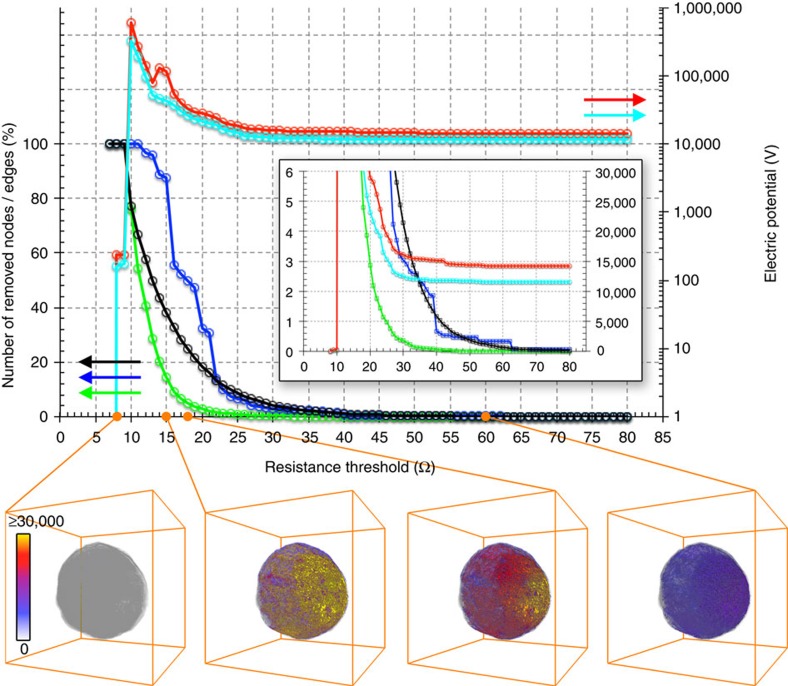
Simulated gradual ageing of the catalyst particle's pore network. The response of the pore network of the FCC particle to pore blocking by the poisoning metals was tested by applying a series of resistance thresholds to remove pore channels (edges of the pore network) with resistance values above the respective threshold. The top part of the figure reports the number of edges that have been removed (in percentage of all edges of the network, black line), the number of source nodes (nodes in Zeolite domains, green line) as well as the number of sink nodes (nodes at the surface, blue line) that have been disconnected from the network (in percentage; a node is removed when there is no edge of the network left connecting to it), and the maximum (red) and average (cyan) pseudo-voltage determined for the network after the edges have been removed. The inset shows a zoom of the plots using a linear scale for the electric potential. In the lower part, a virtual cut through the 3D potential distribution as determined for the network after the pore channels have been removed is reported for the resistance thresholds 8, 15, 18 and 60 Ohm. The total particle volume is indicated in transparent grey. See also Supplementary Movie 2.

The result of this network robustness test is displayed in Fig. 4 and Supplementary Movie 2 showing its high stability for resistance thresholds down to 29 Ohm, which removes less than 5% of all resistors and hardly affect sink and source nodes. For thresholds below 29 Ohm (that is, after a certain point in time or strength of metal blocking), the number of removed resistors, source and sink nodes becomes significant and both maximum and average potential start to rapidly increase to values above 1E5 volts, indicating an increasing number of pores with large pseudo-voltage. In analogy to a pressurized porous medium, these regions are ‘under high pressure' due to the restricted flow (increased resistance) between source and sink nodes of the network. As a direct consequence of the higher Fe, Ni and Ca concentrations at the particle surface more surface (sink) than source nodes are removed. This effectively simulates the collapse of the pore structure in the surface of FCC catalyst particles caused by vitrification, which was suggested to be related to the deposition of elevated amounts of Fe and Ca^22^,^26^,^49^. Below threshold values of ∼20 Ohm, source nodes also start to be decimated, indicating that more and more zeolite domains are disconnected from the network, therefore becoming completely inaccessible.

These effects, which simulate a gradual ageing of the pore network by metal pore clogging, are also reflected in the 3D pseudo-voltage distributions calculated after applying different thresholds (Fig. 4 bottom, Supplementary Movie 2): below ∼24 Ohm sub-regions with a potential beyond 2E4 volts start to appear, which dramatically grow when more and more pore channels of the network become blocked. When removing pore channels the calculated potential first increases, indicating a ‘pressure' rise in the system because most of the source nodes are still present but only a few connections to the surface remain open. Ultimately, after too many pore channels are blocked (removed) at threshold values below 10 Ohm, network and potential distribution collapse as most connections between source and sink nodes are no longer available for transport.

These results show that due to its high degree of interconnectivity, the macro-pore network of the FCC particle is surprisingly robust against pore blocking. Even when a large number of pore channels are blocked some detouring paths remain open, maintaining accessibility of the zeolite domains. These remaining paths of the macro-pore network have the lowest resistance values of the resistor network and can therefore be considered as true ‘highways' of the pore network, because they either have a larger pore diameter than most other pore channels, or are minimally affected by metal deposition because they are located in regions of lower metal deposition, or both.

The presented model for simulating and quantifying the virtual ageing of the catalyst pore network is based on and fully utilizes a correlative data set that combines high-resolution morphological information with chemical information, that is, the 3D relative concentration distribution of multiple poisoning metals. On the basis of the measured macro-porosity (pore geometry and connectivity) and the degree of metal poisoning, we established an accessibility metric for whole, individual FCC particles. We further quantified an individual particle's response to metal deposition and related morphological changes that happen during catalyst ageing. This information together with the possibility to predict the collapse of the catalysts mass transport ability at specific levels of metal deposition is crucial for an improved rational design of heterogeneous catalysts. However, the developed analytical approach is generally applicable to studies of dynamic changes in pore networks of hierarchically structured functional materials, including batteries, fuels cells and optical devices.

## Methods

### X-ray nano tomography

Full-field transmission hard X-ray microscopy was performed at beamline 6-2C of the Stanford Synchrotron Radiation Lightsource at the SLAC National Accelerator Laboratory. Details of the experimental setup can be found elsewhere^50^. X-ray nano tomography was conducted at 8,500 eV with an angular step size of 0.5 degrees over a range of 180 degrees, enabling a high-quality reconstruction of the 3D structure of the FCC particle using the standard Filtered Back Projection algorithm. The effective 3D spatial resolution was estimated to be 98.2 nm by Fourier Shell Correlation analysis^51^ of the tomography data. The evaluation of the pore throat distribution was performed using a numerical solid expansion method^45^, which indicated that the majority of the macro pore throats were at a size of ∼320 nm. This is clearly larger than the achieved effective spatial resolution, which therefore is sufficient for resolving those macro pore channels in the 3D architecture of the FCC particle that act as ‘highways' for both molecules entering the catalyst particle and product molecules leaving it. More details can be found in the section ‘Hard X-ray full-field transmission X-ray microscopy' in the Supplementary Methods.

### Micro X-ray fluorescence tomography

To improve sensitivity for elemental detection, we conducted hard X-ray μ-XRF imaging experiment at beamline 2-3 of the Stanford Synchrotron Radiation Lightsource at the SLAC National Accelerator Laboratory. A double crystal monochromator was used to select X-rays at 8,500 eV that were further focused by a K-B mirror to a spot size of 2 μm, which was then used for raster scanning the sample in fly scan mode with a dwell time of 100 ms for each pixel. The two-dimensional XRF maps were evaluated using fluorescence peak fitting of every single pixel XRF spectrum using PyMca^52^ to quantify the elemental concentrations. This resulted in a set of two-dimensional μ-XRF maps (one for every element) at each angle, which was then reconstructed into 3D elemental distributions applying an Algebraic Reconstruction Technique available in the software package TXM-Wizard^53^. More details can be found in the section ‘Hard X-ray fluorescence tomography' in the Supplementary Methods.

Numerical corrections of the 3D elemental distribution were carried out to overcome artifacts caused by self-absorption effects (see Supplementary Methods: Correction of self-absorption effects in micro XRF tomography ). The obtained 3D matrices were resampled to a pixel size of 64 × 64 × 64 nm^3^ using cubic interpolation to match the high resolution nano-TXM data (see Supplementary Methods: Influence of the XRF resolution and Supplementary Figs 5 and 6). The obtained 3D XRF maps allow analysing correlations among these elements in a quantitative manner. The degree of spatial bimodal elemental correlation offers unique insight into the poisoning metal accumulation mechanisms. Using the entire 3D data set in this correlation analysis ensured that a sufficiently large number of data points (voxels) were used, in turn assuring the statistical significance of the results (see Supplementary Methods: Inter-elemental correlation analysis).

Here it is important to note that in this correlative imaging experiment, the single FCC particle under study was loaded into a Kapton capillary that was mounted in the same sample holder, which could directly be transferred between the two beamlines, allowing straightforward registration of the 3D data.

### Resistor network model

The resistor network was generated by taking advantage of the correlative nature of the micro XRF data and the nano TXM data. In our model, every node of the pore network is connected to its neighbouring nodes via a resistor with a value *R* (in Ohm) that is determined via the formula:


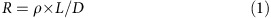


where *L* (in nm) is the length of the pore channel, *D* (in nm^2^) its average cross section, and *ρ* the specific resistance. In the first calculation, *ρ* was set to 1 (Ohm × nm) for all points of the pore network to exclusively assess the effect of the macro pore geometry as reconstructed from nano-TXM data. The equivalent resistance was then determined for each source node of the network, that is, nodes of the network in regions of highest La concentrations, as measured by μ-XRF. This equivalent resistor value of each source node was obtained via nodal analysis, where each source node was defined as a current source of 1A and all sink nodes (surface nodes) were set to be at 0 V (ground). In the second step, the nodal analyses was performed using the relative elemental concentrations of poisoning metals detected by μ-XRF in each point of the pore channel to increase the specific resistance *ρ* of the resistor that represents the respective pore channel. This allowed establishing a relative measure of the ‘resistance' each metal poison poses to molecules travelling from source to sink nodes, assuming that higher metal concentration means stronger pore blockage. The result of the nodal analysis of these two resistor networks provides a pseudo-electric potential in every node of the network, which in turn allows determining an equivalent resistor value between all sink nodes (at ground) and each source node of the network following Ohm's law. More details can be found in the sections ‘Resistor network model' and ‘Re-evaluating the interconnectivity of the pore network' in the Supplementary Methods.

### Data availability

All data that support the findings of this study are available from the corresponding authors upon reasonable request.

## Additional information

**How to cite this article**: Liu, Y. *et al*. Relating structure and composition with accessibility of a single catalyst particle using correlative 3-dimensional micro-spectroscopy. *Nat. Commun.* 7:12634 doi: 10.1038/ncomms12634 (2016).

## Supplementary Material

Supplementary Information Supplementary Figures 1-6, Supplementary Methods and Supplementary References

Supplementary Movie 1 Correlative X-ray microscopy data. The movie shows the data recorded by μ-XRF and nano-TXM tomography. First the total particle volume as measured by 3D μ-XRF is presented, which is then segmented into the different constituents (V, La, Fe, Ni, Ti, Ca) using different colors. Then the correlated high-resolution nano-TXM data is shown (starting at second 24). At second 36 the movie zooms in on an arbitrarily selected subvolume of the catalyst particle entering the pore space. The camera follows a path through the pore network represented by white lines that connect nodes of the network (white spheres). After that the same tracking shot is repeated including the μ-XRF data showing the distribution of the poisoning metals Ca, V, Ni, and Fe, where the intensity of the color is based on the relative concentration of the element.

Supplementary Movie 2 Virtual ageing of the pore network of the catalyst particle. The movie shows the evolution of the 3D voltage distribution with decreasing resistance threshold. In the top part the respective threshold used to remove pore channels with resistance values above the threshold is indicated by a green dot. The graph also shows the maximum voltage of determined for this threshold. Additional indicators (number of removed edges, sink nodes, and source nodes) are provided in the right part of each frame. The total particle volume is indicated in transparent gray.
