# Supplementary material for: Relating structure and composition with accessibility of a single catalyst particle using correlative 3-dimensional micro-spectroscopy
Source: Nat Commun. 2016 Aug 30;7:12634. doi: 10.1038/ncomms12634 (PMC5013607; doi:10.1038/ncomms12634)
Supplement: Supplementary Information — Supplementary Figures 1-6, Supplementary Methods and Supplementary References [file ncomms12634-s1.pdf]

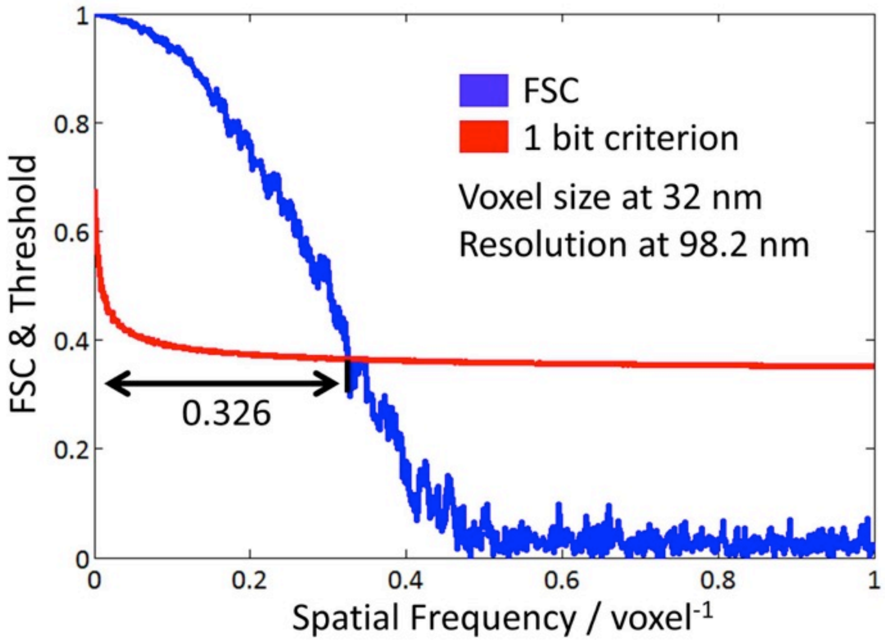


**Supplementary Figure 1. Fourier Ring correlation (FRC) evaluation of the nano-TXM tomography data.** The result indicates an effective 3D spatial resolution of about 98.2 nm (0.326 reciprocal pixels of 32 nm size) as determined by the 1 bit criterion.

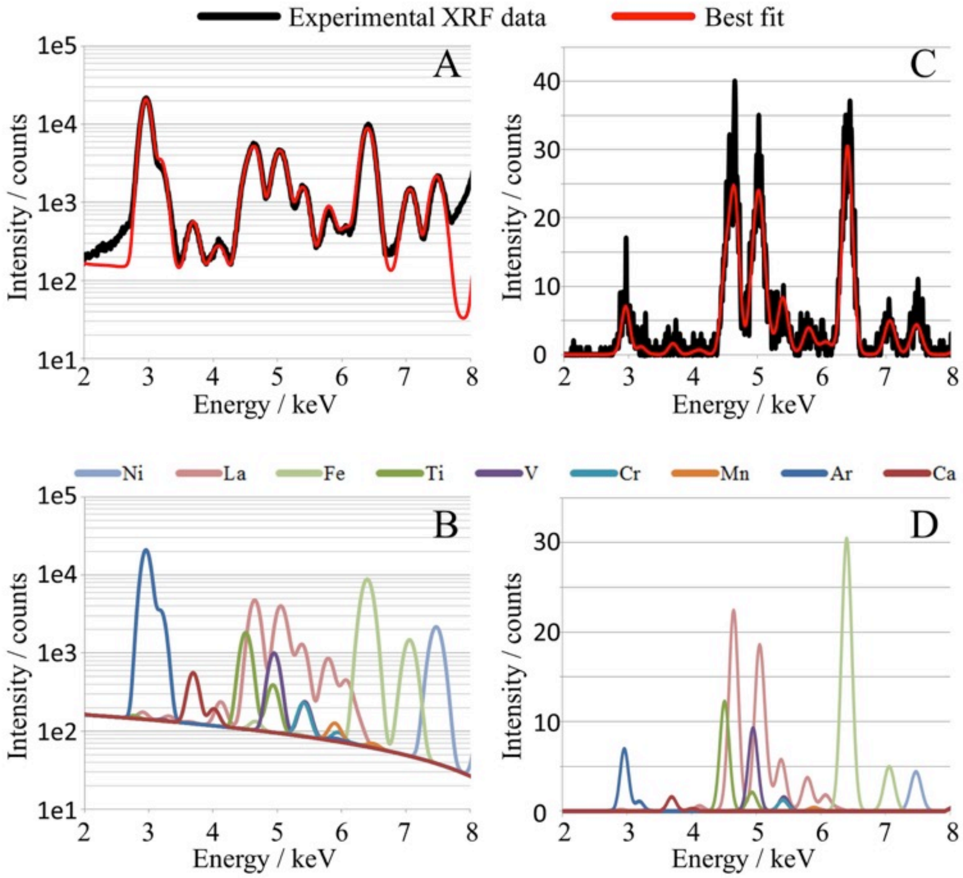


**Supplementary Figure 2. Experimental XRF data and corresponding best fits.** Panels (**A**) and (**C**) show the overall comparison of the experimental data and the best fit of the bulk signal (sum of the XRF signal over the entire particle) and single pixel signal (pixel size at ~2 micron), respectively. Panels (**B**) and (**D**) show the fitting model, i.e. the contributions from all emission lines of the different elements present in the sample. Panels (**A**) and (**B**) are plotted in logarithmic axis for better visualization of the data.

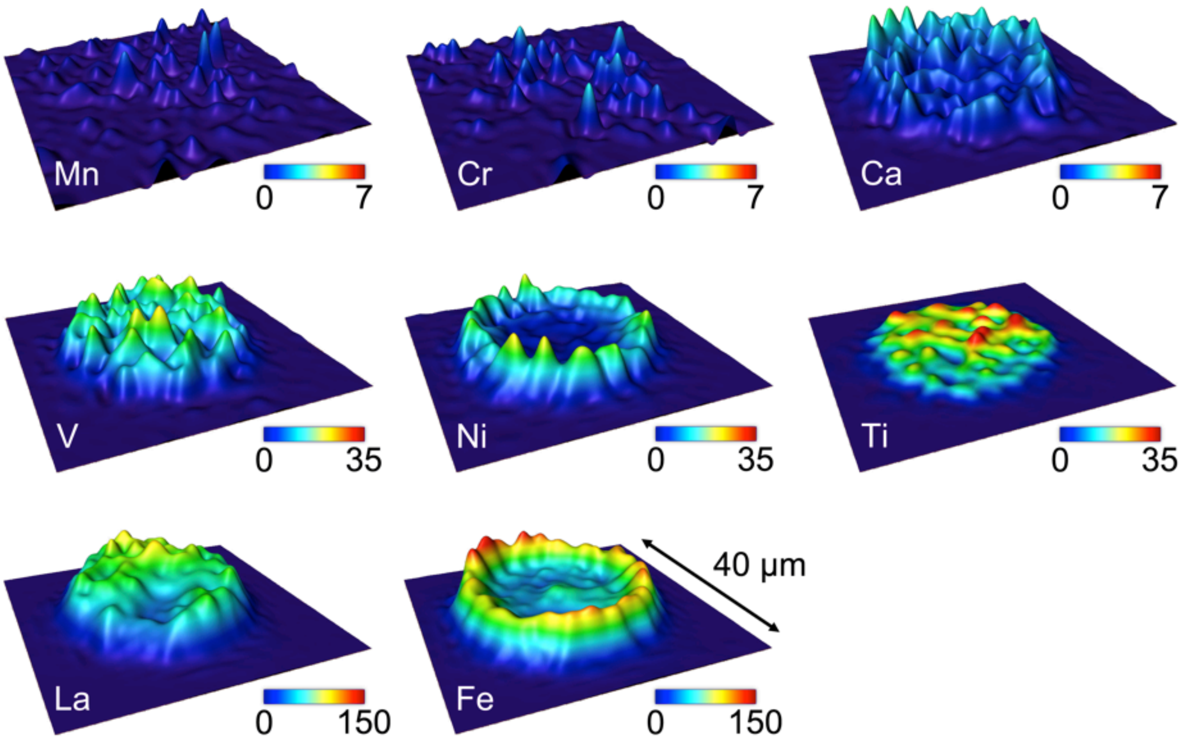


**Supplementary Figure 3. Reconstructed XRF intensities in the central slice of the 3-D data.** Mesh plots displaying the elemental distribution as recorded for the central slice of the FCC particle under study. The displayed XRF peak intensities have been scaled differently for better visualization as indicated by the corresponding color scale bars.

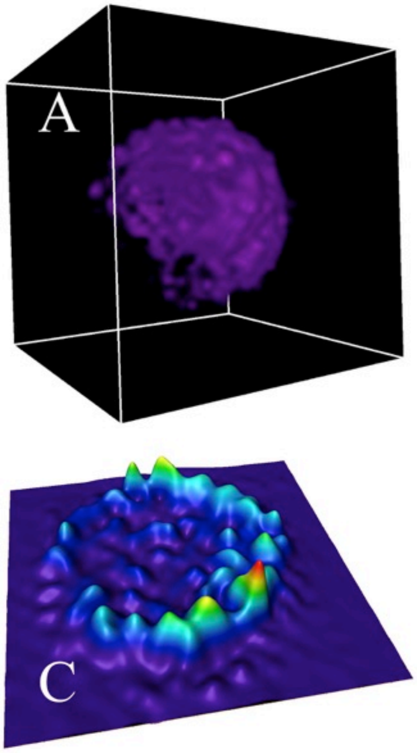


**Supplementary Figure 4. Reconstructed Ca distribution with and without self-absorption correction.** Panels (**A**) and (**B**) show the 3D rendering of the Ca distribution without (**A**) and with (**B**) applying the self-absorption correction. Corresponding central slices through the 3-D data are displayed in panels (**D**) and (**C**).

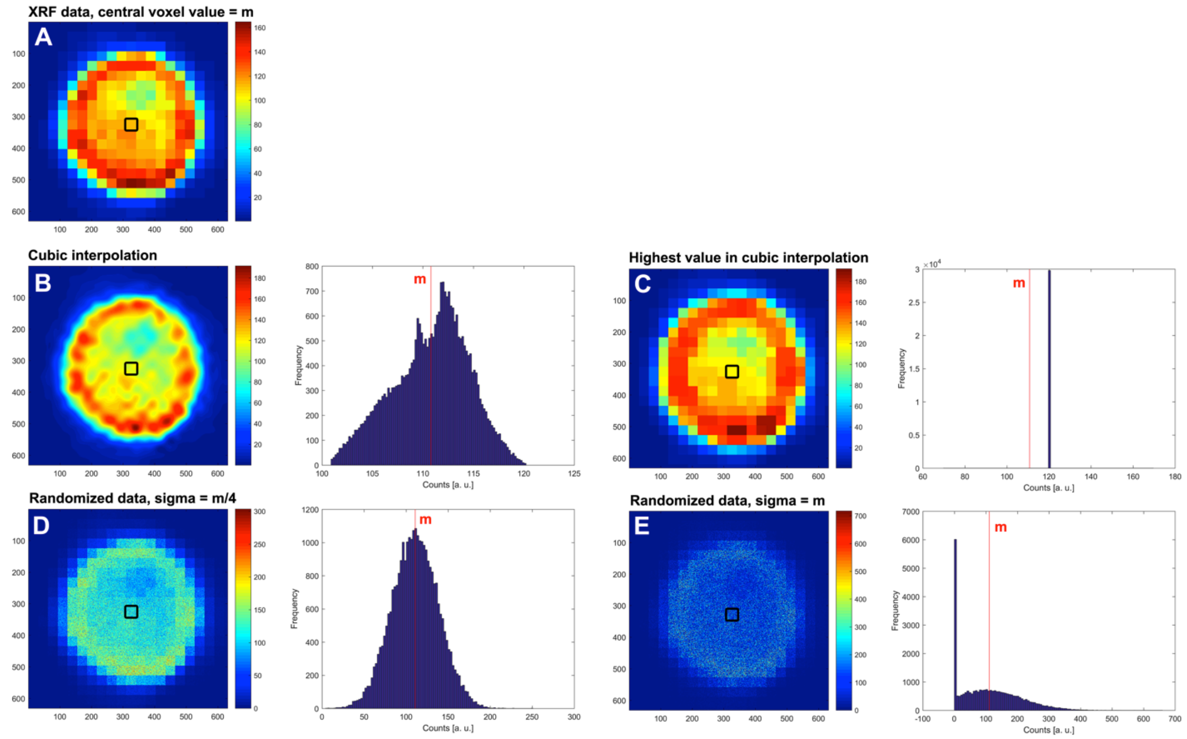


**Supplementary Figure 5. Generated high-resolution 3D XRF data sets using different distribution functions within each low resolution XRF voxel.** (**A**) Central slice through the (self-absorption corrected, summed) XRF data. The black square indicates the central voxel having a value of m, which was measured by the XRF instrument and can be interpreted as the mean value of the unknown high resolution intensity distribution within this voxel. Panel (**B**) shows the same slice through the data obtained using cubic interpolation together with the histogram of the high resolution intensities within the central voxel. The vertical red line indicates the value m. In (**C**) all high resolution voxels within each low resolution voxel have been set to the same value, namely the highest value found by cubic interpolation. Panels (**D**) and (**E**) display randomized data, where the high resolution intensity distribution was described by a normal distribution centered at m and with a sigma of m/4 and m respectively. In the latter case negative values where set to 0.

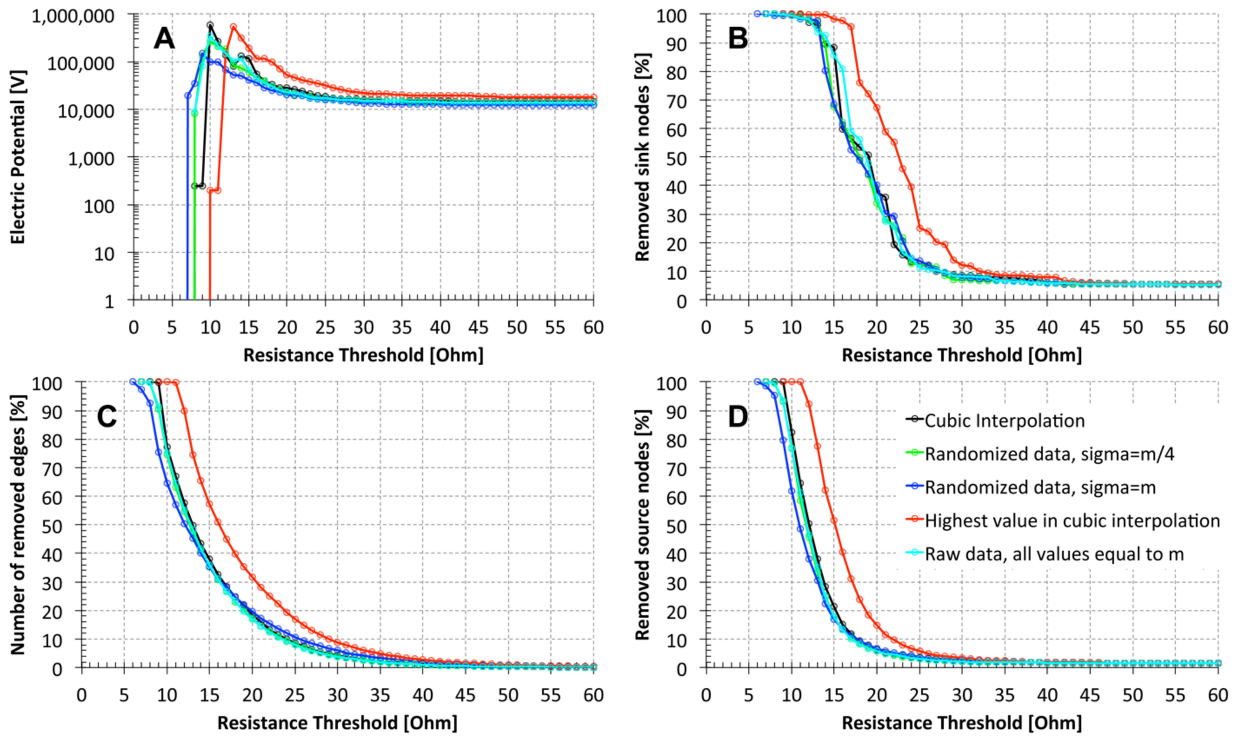


**Supplementary Figure 6. Influence of the high resolution distribution of the elements within each low resolution voxel on the pore network stability simulation.** The response of the pore network of the FCC particle to pore blocking by the poisoning metals was tested by applying a series of resistance thresholds to remove pore channels (edges of the pore network) with resistance values above the respective threshold. The simulation was performed separately for each of the five data sets displayed in Supplementary Figure 5. Panel (**A**) reports the maximum pseudo-voltage determined for the network after the edges have been removed. In (**B**) the number of sink nodes (nodes at the surface) that have been disconnected from the network (in percent; a node is removed when there is no edge of the network left connecting to it) is plotted and panel (**D**) reports the same for source nodes (nodes in Zeolite domains). The plot in (**C**) shows number of edges that have been removed (in percent of all edges of the network).

**Supplementary Methods:**

**Hard X-ray full-field transmission X-ray microscopy**

Hard X-ray full-field transmission X-ray microscopy was performed at beamline 6-2C of the Stanford Synchrotron Radiation Lightsource at the SLAC National Accelerator Laboratory. Details of the experimental setup can be found elsewhere[1]. X-rays at 8500 eV were selected using a double Si(111)-crystal monochromator for illumination of the sample. Tomography data was collected from 0 to 179.5 degrees with an angular step size of 0.5 degrees, which enabled a high quality reconstruction of the 3D structure of the FCC particle using the Filtered Back Projection (FBP) algorithm. Two horizontally adjoined fields of view (FOV) of 30 x 30 $\mu m^2$ were acquired at each viewing angle extending the total FOV to cover the entire particle with a diameter of about 40 μm. The voxel size of the 3-D reconstruction was at $32\times32\times32$ $nm^3$, and was further binned to a size of $64\times64\times64$ $nm^3$ in order to mitigate the requirement of large scale computing resources during quantification and modeling of the complex 3-D pore network and to account for the achieved effective 3-D spatial resolution. The effective 3-D spatial resolution was estimated by performing a Fourier Shell Correlation analysis[2] of the tomography data: the full tomography data set (0 to 179.5 degrees) was split into to two subsets such that the first data set contained images recorded at odd projection angles and the second images recorded at even projection angles. The FRC curve displayed in Supplementary Figure 1 shows that the effective 3D spatial resolution determined using the 1 bit criterion was 98.2 nm (0.326 reciprocal pixels of 32 nm size). TXM data processing was performed using TXM-Wizard[3], an in-house developed software package. The evaluation of the pore throat distribution was performed using a numerical solid expansion method[4], indicating that the majority of the macro pore throats were found at a size of ~320 nm. This is clearly larger than the achieved effective spatial resolution,

which therefore is sufficient for resolving those macro pore channels in the 3D architecture of the FCC particle that act as “highways” for both crude molecules entering the catalyst particle and product molecules leaving it.

**Hard X-ray fluorescence tomography**

The hard X-ray μ-XRF imaging experiment was performed at beamline 2-3 of the Stanford Synchrotron Radiation Lightsource at the SLAC National Accelerator Laboratory. A double crystal monochromator was used to select X-rays at 8500 eV that were further focused by a K-B mirror to a spot size of 2 μm, which was then used for raster scanning the sample. Data acquisition of μ-XRF images was performed using the software package SMAK[5]. More details about the experimental setup can be found elsewhere[6].

Raster scans were performed in fly scan mode with a dwell time of 100 microseconds for each pixel to improve the data acquisition speed. Tomography was performed with angular steps of 2 degrees. The 2D XRF maps recorded at each angle were evaluated using fluorescence peak fitting of every single pixel XRF spectrum using PyMca[7]. A representative spectrum is presented in Supplementary Figure 2, showing experimental data and the fit used to evaluate the individual contributions from different elements of interest (peak areas of the fit) providing information about the elemental composition of the sample. This resulted in a set of 2D μ-XRF maps (one for every element) at each angle. Supplementary Figure 3 displays the mesh view of the central slice in the x-y plane through the reconstructed 3-D XRF tomography data for each evaluated element after correction for self-absorption effects (see below). These plots show that the recorded XRF intensities, i.e. the detected concentrations for Mn and Cr are close to the limit of detection, especially because the fluorescence lines of these two elements overlap with the L-lines of La in the XRF spectrum (see Supplementary Figure 2), which complicates a precise quantification for

those two metals. For Ca on the other hand a clear peak is visible in the XRF spectrum, confirming its presence, although at much lower concentrations than the other evaluated metals. Although the V K-lines overlap with the La L-lines in the XRF spectrum, a good deconvolution of their signals was possible, confirmed by the fact that V and La are not perfectly correlated (see Figure 1), i.e. regions in the particle exist that show elevated V concentrations but low amounts of La.

The 2D XRF maps generated by PyMca[7] were then reconstructed into 3D elemental distributions applying an Algebraic Reconstruction Technique (ART) available in the software package TXM-Wizard[3]. The obtained 3-D matrices were resampled to a pixel size of 64×64×64 $nm^3$ using cubic interpolation in order to match the high resolution nano-TXM data. Here it is important to note that in this correlative imaging experiment, the single FCC particle under study was loaded into a Kapton capillary that was mounted in the same sample holder, which could directly be transferred between the two beamlines, allowing straightforward registration of the 3-D data.

Furthermore, in order to remove/minimize artifacts in the XRF data caused by the self-absorption effect, we simultaneously recorded the transmitted X-ray intensity during the raster scan (further denoted as the STXM data) and developed an algorithm to perform an effective self-absorption correction (see below).

**Correction of self-absorption effects in micro XRF tomography**

The so-called self-absorption effect is a well-known challenge in the evaluation of XRF tomography data. In order to remove/minimize artifacts caused by this effect, we also recorded the transmission X-ray intensity in the μ-XRF raster scan (STXM data) and developed an algorithm to perform the correction.

The μ-XRF signal emitted from a certain voxel within the 3D structure has to travel through the sample before the XRF detector can record it. This causes absorption of the XRF signal by the sample itself ('self-absorption') that depends on several factors, including the relative position of the voxel, the 3-D density distribution, and the 3-D elemental distribution in the sample. An absolute correction of this effect is therefore complicated by the complexity of composition and heterogeneity of the studied sample.

In this work, we developed a simplified correction procedure using *a priori* knowledge of the experimental setup geometry, the average bulk density of the sample, the average elemental composition of the FCC particle, and the energy of the XRF emission lines. First the recorded STXM data was used to perform an initial tomography reconstruction, which provided the distribution of the 3D optical density recorded at 8500 eV. Then, putting together all *a priori* information, we constructed a 3D correction matrix by cumulatively summing the 3D optical density map along the direction from the sample to the detector and further scaled it by an energy dependent factor that takes into account the elemental composition of the sample. This approach effectively calculates the average X-ray absorption of the emitted radiation for all X-ray fluorescence energies and all voxel positions in the sample using the X-ray absorption coefficient μ and Beer-Lambert's law.

The effect of the above-explained method for self-absorption correction is illustrated in Supplementary Figure 4 by means of a direct comparison of the Ca distribution displayed with and without self-absorption correction. The self-absorption effect is strongest for Ca because the Ca XRF emission line has the lowest X-ray energy. The improvement is obvious as the missing part in Supplementary Figure 4A becomes clearly visible in Supplementary Figure 4B.

This approach provides a simple and fast method for correcting self-absorption effects but is limited by the assumption that every voxel contains the same element concentration ratios. Voxels that contain very different ratios will have a different value of the X-ray absorption coefficient μ, for example in the unlikely case that a voxel contains pure Ni. However, because self-absorption is directly proportional to the length of the radiation path within the material (following Beer-Lambert's law), significant self-absorption effects are only expected for greater sample thicknesses, i.e. for a path through many voxels. This in turn means that the stronger this effect becomes, the more likely it is that the actual absorption coefficient for the total radiation path length becomes the average absorption coefficient of the material. In the specific case of FCC particles the above assumptions are further supported by the fact that more than 90% of the material consists of Al, Si and O[8], which means that the absorption coefficient is dominated by elements that are fairly homogenously distributed[9] and are further not strongly absorbing at the X-ray energies of interest (i.e. energies equal and larger than the Ca K-alpha line at 3.69 keV).

A more complex (and computationally expensive) approach for correcting the μ-XRF tomography data would be to modify the above described correction into an iterative method. The correction achieved in the iterations leads to an improved distribution of elements of interest and thus can affect the amount of self-absorption for each individual voxels. The secondary absorption is another factor that needs to be taken into consideration in the iterations, due to the complexity in the elemental composition. However, we expect the amount of improvement that can possibly be achieved by this complicated iterative method to be very limited for this study, because of the small size of the particle relative to the X-ray absorption length.

**Inter-elemental correlation analysis**

As discussed in the section above, distributions of multiple elements were recorded simultaneously during XRF mapping, and were further quantified by performing XRF peak fitting. The obtained results allow analyzing correlations among these elements in a quantitative manner. The degree of spatial bimodal elemental correlation offers unique insight into the poisoning metal accumulation mechanisms. Using the entire 3-D data set in this correlation analysis ensured that a sufficiently large number of data points (voxels) were used, in turn assuring the statistical significance of the results.

In these plots voxels that contained no or very low elemental concentrations of both elements have been removed to avoid that this large number of 'empty' voxels dominates the correlation. Specifically, if a voxel contained less than 5% of the maximum concentration of element A and B respectively, it was not considered in the bimodal spatial correlation of A and B. This ensured that voxels with low or no concentrations of element A but high concentrations of element B (and *vice versa*) were not removed.

**Resistor network model**

In our model every node of the pore network is connected to its neighboring nodes via a resistor with a value R (in Ohm) that is determined via the formula:

$$R = \rho \times L / D \quad [1]$$

where L (in nm) is the length of the pore channel, D (in $nm^2$) its average cross section, and ρ the specific resistance. In the first calculation ρ was set to 1 (Ohm × nm) for all points of the pore network to exclusively assess the effect of the macro pore geometry as reconstructed from nano-TXM data. The equivalent resistance was then determined for each source node of the network, i.e. nodes of the network in regions of highest La concentrations (≥ 60% of the maximum La

intensity), as measured by μ-XRF. This equivalent resistor value of each source node was obtained via nodal analysis, where each source node was defined as a current source of 1 A and all sink nodes (surface nodes) were set to be at 0 V (ground). The 60% threshold for La intensities was used to isolate regions of highest La intensity while accounting for the limited resolution of the XRF instrument and is somewhat arbitrary. It is known that zeolite domains in FCC catalyst particles have sizes between a few hundreds of nanometers to 1-2 microns[10-12], which are below the resolution achieved in the XRF data. This lack in resolution blurs the distribution of zeolite domains and the XRF data can therefore be understood as probability map for finding pure zeolite domains. The highest probability of finding a pure zeolite domain correlates with the highest La intensities and was therefore used to identify zeolite domains.

In the second step the nodal analyses was performed using the relative elemental concentrations of Fe, Ni, Ca, and V as detected by μ-XRF in each point of the pore channel to increase the specific resistance ρ of the resistor that represents the respective pore channel. Specifically, ρ of each path connecting two nodes (i.e. one resistor) was determined as 1 + the sum of the average elemental concentrations of Fe, Ni, Ca, and V along the path - analogue to a conducting wire with length L, cross section D, and a specific resistance that depends on the elemental concentrations along the pore channel. In this way every connection ('edge') of the network was assigned a unique (pseudo-electrical) resistance value that was based on the respective metals 3D concentration distributions. This allowed establishing a relative measure of the 'resistance' each metal poison poses to molecules traveling from source to sink nodes, assuming that higher metal concentration means stronger pore blockage.

The result of the nodal analysis of these two resistor networks provides a pseudo-electric potential in every node of the network, which in turn allows determining an equivalent resistor

value between all sink nodes (at ground) and each source node of the network following Ohm's law.

In order to quantify and visualize relative changes in particle accessibility, we determined changes in the distributions of pseudo-electrical potential (Figure 3C and H) and equivalent resistance (Figure 3G and I) by computing the difference between normalized distributions obtained with and without considering the presence of the metals Fe, Ni, V, and Ca. Normalized distributions were used to exclusively determine changes in the distribution, because these changes show how the network responds to local changes in resistance due to the presence of the metals.

In order to test the response of the pore network to complete pore blocking (i.e. the removal of connections between nodes) we established and evaluated 73 resistor networks by applying 73 thresholds ranging from 8 to 80 Ohm. Resistors with resistance values above the threshold were removed from the network and the network was re-evaluated with respect to interconnectivity (e.g. considering the formation of disconnected sub-networks, see below). This range of threshold values was based on the maximum resistance value (149 Ohm) found for the pore network considering all metals and the fact that a threshold of 80 Ohm removed only 0.022% of all edges of the pore network (43 of 193400), while all edges were removed at a threshold of 7 Ohm.

Here it is important to note that we used the resistivity value as determined by equation 1 and not simply the sum of metal concentrations in order to take the specific pore geometry into account. More specifically, because specific resistance $\rho$ and pore channel cross section D are anti-correlated for constant resistance narrower pores are removed at lower metal concentrations than larger ones.

**Re-evaluating the interconnectivity of the pore network**

When removing resistors with resistance values above the threshold from the resistor network, the whole network has to be re-evaluated with respect to interconnectivity because removing connections in a network can lead to the formation of isolated sub-networks or nodes. These individual nodes or sub-networks of interconnected nodes are disconnected from the main network, and may or may not continue to contribute to the mass transport through the network. This in turn can significantly influence the calculated potential distribution, especially when many sub-networks are created at lower resistor thresholds (i.e. when many resistors are removed). Three possible situations have to be considered:

a) Individual nodes or sub-networks that are connected to one or more source nodes but no longer to the surface (one or more sink nodes); the potential of these nodes cannot be determined, but being connected to source nodes, these regions have very high voltages (they are 'under high pressure'). In order to visualize these regions, which are excluded from mass transport, they were set to the maximum voltage determined for the remaining system.

b) Individual nodes or sub-networks that are no longer connected to source nodes. These regions are at 0 V pseudo-electrical potential (independent of being connected to sink nodes).

c) Regions that are no longer part of the main (largest) network, but are still connected to at least one source and one sink node. These sub-networks are available for mass transport and are therefore included in the calculation of the potential distribution.

**Influence of the XRF resolution**

The developed resistor network model is based on the correlation between lower resolution micro-XRF and higher resolution nano-TXM data. As shown schematically in Figure

2A, there are 31x31x31 high-resolution voxels of 64x64x64 $nm^3$ (nano-TXM data) within each one of the lower resolution voxels of ~2x2x2 $\mu m^3$ (micro-XRF data grid; more precisely voxels of 1984nm edge length). In order to correlate micro-XRF and nano-TXM data (see section 'Hard X-ray fluorescence tomography') we applied commonly used cubic interpolation of the micro-XRF data to ensure that the two data sets have identical voxel sizes. This was implemented in order to avoid the 'hard edges', i.e. a sudden change in the metal concentration among adjacent voxels (see Supplementary Figure 5A), which is an artifact caused by the limited spatial resolution in the micro-XRF measurement and is not expected to exist in the sample. The effect of cubic interpolation is demonstrated in Supplementary Figures 5A and 5B. However, as the actual high resolution 3D distribution of the elements within each one of the low resolution micro-XRF voxels is unknown, one could think of different possible fine-length-scale metal distributions having the same average value, the latter being the quantity that is in fact acquired through the micro-XRF measurement.

In order to assess the influence and related uncertainty of the unknown high-resolution distribution of the elements within each low-resolution voxel, we repeated the entire pore network analysis several times, each time using a different distribution function. Supplementary Figure 5 shows the low resolution XRF data (Supplementary Figure 5A, displayed is the sum of the element specific XRF counts; see section 'Resistor network model'), the data obtained by cubic interpolation (Supplementary Figure 5B), the data set where all high resolution voxels within each low resolution voxel were set to the highest value obtained by cubic interpolation (Supplementary Figure 5B), and two randomized data sets, using a normal distribution of values within each low resolution voxel (Supplementary Figures 5D and 5E). In each data set the central voxel (indicated by the black square) was inspected as an example to show the effect of the

different distribution functions applied. For the micro-XRF data set (Supplementary Figure 5A) each low-resolution voxel has one value m, which was experimentally measured by the micro-XRF instrument and can be interpreted as the mean value of the intensity distribution of the corresponding high resolution voxel group. After cubic interpolation the same voxel now contains 31x31x31 high-resolution voxels with an intensity distribution shown by the map and the histogram in Supplementary Figure 5B. The value m is indicated by the vertical red line in the histogram. A known effect in (monotone) cubic interpolation is 'overshooting', which can cause the signal to exceed its steady state value. In our application this effect can produce count rates in some interpolated voxels that are slightly higher than the values found in the original low-resolution voxels. This is visible when inspecting the range of the color bars in Supplementary Figures 5A and 5B. In order to inspect the effect of an increased count rate we therefore also generated a data set where each high-resolution voxel was set to the highest count rate generated by cubic interpolation (Supplementary Figure 5C). This effectively simulates a low resolution data set with a higher average value than the one measured by micro-XRF (see histogram in Supplementary Figure 5C) and therefore allows to assess a) the influence of using data that was not 'smoothed' by cubic interpolation and b) the effect of higher metal concentrations on the performed resistor network modeling. Finally, in order to test how the unknown 3D intensity distribution of the metals within each low resolution voxel affects the resistor network modeling, we randomized the distribution within each one of the low resolution voxels using a normal distribution centered at the value m and with a sigma of m/4 and m, respectively. The results of this randomization are displayed in Supplementary Figures 5D and 5E. Here negative intensities (visible e.g. in the data set generated using a large sigma, Supplementary Figure 5E) are set to 0, i.e. simulating voxels without any metals. It is important

to note that for these synthetic data sets shown in Supplementary Figures 5B, 5D, and 5E the average XRF intensity value over the low-resolution voxel (containing 31x31x31 high resolution voxels) always equals the one determined by the measurement (i.e. the voxel values m shown in Supplementary Figure 5A). In the voxels where the negative values are set to be zero (Supplementary Figure 5E) the agreement between the artificial and the experimental data was assured by changing all the positive values accordingly compensating for the large number of high-resolution voxels containing zero intensity. In other words, each of the three artificial data sets displayed in Supplementary Figures 5B, 5D, and 5E could, theoretically, represent the real intensity distribution in the sample, which would results in the same experimental result in the micro-XRF measurement as the one reported in Supplementary Figure 5A. However, we want to point out that a purely random intensity distribution as shown in Supplementary Figures 5D and 5E, i.e. an element distribution that consists almost exclusively of highly localized, 64x64x64 $nm^3$ sized 'hot spots' is rather unlikely. Therefore these data sets can be used as a benchmark to assess how strongly the unknown high-resolution metal distribution within each low-resolution micro-XRF voxel would influence the resistor network modeling.

Each of the five data sets shown in Supplementary Figure 5 was used in a separate resistor network simulation. (The data set shown in Supplementary Figure 5B is the one that was presented in the main text of the manuscript.) The four panels in Supplementary Figure 6 show the response of the pore network of the studied FCC particle to the pore blocking caused by the accumulation of the poisoning metals. The response was evaluated by applying a series of resistance thresholds (at 60 and lower with step size of 1) to remove pore channels (edges of the pore network) with resistance values above the respective threshold. The results clearly show that for the data sets that have the same micro-XRF intensity distribution the response of the pore

network is very similar (black, green, blue, and cyan plots in Supplementary Figure 6). As expected the data sets generated by cubic interpolation (black) and using a sigma of m/4 (green) result in a behavior that is most similar to the one using the ‘raw’ XRF data (cyan plot). More specifically, for these three data sets the network collapses at the same threshold value of 7 (where all edges have been removed). The reason for this similarity is based on the fact that the number of removed edges and sink nodes (nodes at the surface of the particle) is almost identical for those data sets. This has to do with the fact that even though the values of high-resolution voxels have been randomized within each coarse micro-XRF voxel, the highest metal concentrations, which play a dominant role in the pore network response and, ultimately, in its collapse, are still found in the surface regions of the particle. This observation is also confirmed by inspecting the data set with a higher mean value for each low resolution voxel (red plot): as expected the network’s response shows a very similar trend but starts earlier and is completed at a threshold value of 9 instead of 7. Finally, we can also assess the behavior of a highly non-uniform data set generated using a random normal distribution with a sigma value of m (the blue plot). This data set is interesting as it also provides feedback on the influence of the pore network morphology, which is now ‘tested’ by using fewer, highly localized ‘hot spots’ of metal concentration that will now block the pore network at fewer locations, but at higher thresholds. A highly interconnected network like the one we observed in the studied FCC particle is therefore expected to collapse at a later stage, providing alternative pathways when only specific, individual routes are blocked by these highly localized metal concentrations. This is in fact what we observe when inspecting the plots reported in Supplementary Figure 6. While a larger number of edges (pore channels) are removed already at higher thresholds (Supplementary Figure 6C, blue plot down to thresholds of ~20), the collapse of the pore network starts later than

for the other data sets with identical metal distribution at the coarse length scale (black, green, cyan), because alternative pathways are still available for facilitating mass transport. The complete collapse of the network, defined as the threshold at which all edges have been removed, is finally reached at a value of 6, which is lower than in the other scenarios.

These findings are not only in excellent agreement with and support the conclusions drawn on the basis of the evaluation of the data set used in the main text of the manuscript (the data set generated by cubic interpolation), but also provide an effective assessment of a possible uncertainty introduced by the discrepancy in spatial resolution of nano-TXM and micro-XRF data. The results of our simulations using different element distributions within each low resolution micro-XRF voxel show that although the developed resistor network model is clearly sensitive to the differences in the metal distributions, the overall response of a highly interconnected network, such as the one found in the studied FCC particle, is dominated by the distribution of metal concentration at relatively coarse scale that corresponds to the low resolution micro-XRF voxels. As expected, such a highly interconnected network is even more stable against increasingly non-uniform distributions (see for example the data set generated using a value of sigma = m).

We therefore conclude that in the presented case the uncertainty introduced by correlating lower resolution micro-XRF data with higher resolution nano-TXM data through cubic interpolation of the XRF data to match the voxel size does not significantly affect the quantification and overall result of the resistor network modeling. One reason for this relative stability of the network response against different element distributions is related to its high interconnectivity.

Finally we would like to point out that a high standard deviation distribution of metal concentrations within the coarse voxels at ~ 2x2x2 $microns^3$ (as used in the data set shown in Supplementary Figure 5E) is very unlikely in reality. In an industrial reaction unit, the deposition of the poisoning metal on a single FCC particle accumulates over long time periods. As a result, it is more likely that the distributions of the poisoning metals within the FCC particle have low spatial frequency characteristics, i.e. the concentration distribution is varying relatively slowly and without abrupt jumps among adjacent voxels. This statement is supported by recent imaging results showing the Fe distribution in an industrial FCC particle at even higher resolution (<15nm in 2D mapping) than was achieved here using TXM[10].

**Supplementary References:**


1. Liu, Y. *et al.* 3D elemental sensitive imaging using transmission X-ray microscopy. *Anal. Bioanal. Chem.* **404**, 1297-1301 (2012).

2. van Heel, M. & Schatz, M. Fourier shell correlation threshold criteria. *J. Struct. Biol.* **151**, 250–262 (2005).

3. Liu, Y. *et al.* TXM-Wizard: a program for advanced data collection and evaluation in full-field transmission X-ray microscopy. *J. Synchrotron Rad.* **19**, 281-287 (2012).

4. Yang, F. *et al.*, Extraction of pore-morphology and capillary pressure curves of porous media from synchrotron-based tomography data. *Scientific Reports*, **5**, 10635 (2015).

5. Webb, S. The MicroAnalysis Toolkit: X-ray fluorescence image processing software. *AIP Conference Proceedings* **1365**, 196-199 (2011).

6. Korbas, M. *et al.* Localizing organomercury uptake and accumulation in zebrafish larvae at the tissue and cellular level. *Proc. Natl. Acad. Sci. USA* **105**, 12108-12112 (2008).

7. Solé, V.A. *et al.* A multiplatform code for the analysis of energy-dispersive X-ray fluorescence spectra. *Spectrochim. Acta Part B* **62**, 63-68 (2007).

8. Scherzer, J. Designing FCC catalysts with high-silica Y zeolites. *Appl. Catal.* **75**, 1-32 (1991).

9. Yaluris, G., Cheng, W.-C., Peters, M., McDowell, L. T., Hunt, L. Mechanism of fluid cracking catalysts deactivation by Fe. *Stud. Surf. Sci. Catal.* **149***,* 139−163 (2004).

10. Wise, A.M., Nelson Weker, J., Kalirai, S., Farmand, M., Shapiro, D.A., Meirer, F., Weckhuysen, B.M. Nanoscale Chemical Imaging of an Individual Catalyst Particle with Soft X-ray Ptychography. *ACS Catal.* **6**, 2178-2181 (2016).

11 Ristanović, Z., Kerssens, M.M., Kubarev, A.V., Hendriks, F.C., Dedecker, P., Hofkens, J., Roeffaers, M.B.J., Weckhuysen, B.M. High-Resolution Single-Molecule Fluorescence Imaging of Zeolite Aggregates within Real-Life Fluid Catalytic Cracking Particles. *Angew. Chem. Int. Ed.* **54**, 1836–1840 (2015).

12 Sprung, C., Weckhuysen, B.M. Dispersion and Orientation of Zeolite ZSM-5 Crystallites within a Fluid Catalytic Cracking Catalyst Particle. *Chem. - Eur. J.* **20**, 3667–3677 (2014).
